# Annotations

**Published:** 1893-02-18

**Authors:** 


### Annotations.

**Invertebrate Medicine.**

The quantity of knowledge floating about in medical minds and medical books at the present day is overwhelming. It reminds one of the early condition of the earth as assumed by the Mosaic cosmogony. "The earth was without form, and void." Such was the idea of those early minds which perceived the feebleness and inconsequence of formlessness, and the definiteness and power of form and order. The medical earth is to a large extent without form, though it is by no means void. On the contrary, it is full, even to hopeless confusion, of an almost infinite variety of knowledge. "Oh! for a selective and constructive mind," one cannot help exclaiming. Medicine has a career, a mission for the world, but it lacks practical leaders. The practical leader is the man who sees the essential and, separating it from the accidental, combines it into a powerful instrument for the doing of practical work. What a marvellous and mighty instrument of amelioration, of strengthening for the universal body and mind, might be constructed from the *disjecta membra* of medical and scientific knowledge which lie in hopeless incombination all over the civilised world! Here and there a competent mind selects and combines and makes such a powerful instrument as we have described. But he makes it for himself alone; he, in fact, becomes the powerful instrument, and when he dies the instrument dies with him. Such a man gives up to a small and limited clientele what should be given to the whole of medical mankind. As is the one capable selector and combiner, so should every medical man be; and the aim of those who realise the true nature of the career and mission of medicine should be to construct an instrument, a compressed literature, an assemblage of canonical books, gathered from all the medical ages, which should be to the medical world what the Bible has been to the Church. Such an assemblage of canonical books would never grow old. It would make strong and stalwart physicians. It would be a central point round which all new knowledge for all future time would crystallise and accumulate. Where is the constructive mind, or minds? And who has the needed leisure?

**Plumbing and Cholera.**

The Blue Book containing the evidence given before the recent Royal Commission on the Registration of Plumbers sets forth matter of serious import to those communities which congregate in large towns. For example, the Chairman asked (Q. 29), "Whose estimate is it that one-third of the preventable illnesses in the United Kingdom are attributable to defective plumber's work?" The answer was that "Dr. Pridgin Teale, of Leeds" had made the estimate. We do not know on what basis of facts Mr. Pridgin Teale had founded his conclusion, but it is quite certain that he is not the man to have committed himself to such a statement without reasonable justification. Now if this conclusion of an eminent sanitary authority be considered along with statements made at page 21 of the report, much food for rumination will be found. The Chairman asks (Qs. 333-334), and Mr. Thomas Anderton, President of the United Operative Plumbers' Association of Great Britain and Ireland, answers the following questions: "Is it the fact that at that time" (1876) "jerry builders, as they are called, ironmongers, tinkers, painters, undertakers, and others, seeing that a good profit could be made out of plumbing, added plumbing to their other work?" "That is perfectly true." "And apprentices were then taken without any limit, and were expected to learn, perhaps, six or seven different trades?" "Yes." Bad plumbing, according to Mr. Pridgin Teale, is responsible for one-third of the preventable illnesses in the United Kingdom; but, as things are at present, nobody whatever is responsible for bad plumbers. If any private business were conducted as this department of State business is conducted, irretrievable bankruptcy could not be averted for a single twelvemonth. The Plumbers' Company, whose present master is Lord Mayor Stuart Knill, has made, and is making, serious efforts and considerable sacrifices to bring about a better state of things; and the Editor of the *British Medical Journal*, Mr. Ernest Hart, has devoted much energy and intelligence to the question of plumbers' registration. But for some reason adequate motive power seems to be wanting, and practically we are almost as much at the mercy of "jerry builders, tinkers, painters, and undertakers" for our plumbing as we were fifteen or sixteen years ago. The public should, of course, be its own protector in this matter. But the public, as a public, is dull and lethargic to an almost incredible degree. Except the Plumbers' Company, the only persons who seem to be moving at all are here and there a handful of doctors. Now doctors have much to gain by bad plumbing, and much to lose by good. And yet they rouse themselves to action in the public interest, whilst the gross and stupid public lies fast asleep. We use these hard words for the purpose of forcing attention to the subject if possible. It is much to be feared, however, that something a great deal sterner than hard words will be required before anything effectual is done. But that something, we are confident, will not be long wanting. It may be upon us in the shape of a cholera epidemic at almost any moment.

**A Word for Poor Physic.**

"The Humanitarian" is an American periodical which lives in the clouds, and invites all mankind to follow it to those high spheres. Needless to say it is edtited by a woman. Abstract thinking, indeed, seems now to be generally falling into the hands of women. *The Humanitarian* is nothing if not abstract. Its idea of humanitarianism, if we may judge by the subjects discussed in a recent number, is that men and women will never be happy until they are all abstract philosophers; until, in fact, they do as the Irish sergeant asked his ragged regiment of recruits to do, "Stip out of their ranks and have a look at themselves." Most portentous are the titles of the articles in *The Humanitarian.* "Heredity," "Physical Development," "Pedigree Farming," "Child Culture,"—poor children! "The Efficacy of Punishment," and the like. And some of the articles, as becomes philosophic discussions, are most portentously long. Compared with this, physic, the art of amelioration, is mere tinkering. In high humanitarianism there is no place for physic, for amelioration; there is room only for prompt destruction of the "unfit" and high development of the "fit." But is amelioration really such a very sorry business, after all? The man who removes pain and restores damaged men and women to health, though he be a mere "tinkerer" compared with the abstract philosopher, is yet a person who ought to be permitted to live, and, in case of need, to render service even to the abstract philosopher herself. It is somewhat of a worthy thing, too, to moderate the rough edge of poverty, and to "minister to minds diseased." Of course, in a truly humanitarian world there would be no poverty, and "minds" would never need "ministering to," because they would never be "diseased." It seems, indeed, that in the very highest humanitarian world there are no minds at all, but only properly developed "cerebral cortices." But in the meantime, and for the immediate, and quite unworthy present, the idea of "medicine" for all sorts of purposes—of physical healing, of social amelioration—is still serviceable, if only to make embryo humanitarianism tolerable until fully developed humanitarianism can come in.

